# Piperazine-Derived α_1D/1A_ Antagonist 1- Benzyl-N- (3-(4- (2-Methoxyphenyl) Piperazine-1-yl) Propyl) -1H- Indole-2- Carboxamide Induces Apoptosis in Benign Prostatic Hyperplasia Independently of α1-Adrenoceptor Blocking

**DOI:** 10.3389/fphar.2020.594038

**Published:** 2021-01-27

**Authors:** Qing Xiao, Qi-Meng Liu, Ru-Chao Jiang, Kai-Feng Chen, Xiang Zhu, Lei Ma, Wei-Xi Li, Fei He, Jun-Jun Huang

**Affiliations:** ^1^ The Fifth Affiliated Hospital, Key Laboratory of Molecular Target & Clinical Pharmacology and the State Key Laboratory of Respiratory Disease, School of Pharmaceutical Sciences, Guangzhou Medical University, Guangzhou, China; ^2^ Genetics Laboratory of Obstetrics, The Second Affiliated Hospital of Zhengzhou University, Zhengzhou, China; ^3^ College of Chinese Traditional Medicine, Yunnan University of Chinese Medicine, Kunming, China; ^4^ School of Traditional Chinese Medicine, Southern Medical University, Guangzhou, China

**Keywords:** apoptosis, multi-target activities, B-lymphoma Mo-MLV insertion region 1, benign prostatic hyperplasia, α1-adrenoceptor antagonist

## Abstract

Previous studies have indicated that α_1D/1A_ antagonist naftopidil (NAF) suppresses prostate growth by decreasing cell proliferation without affecting apoptosis and prostate volume in benign prostatic hyperplasia (BPH). A NAF-derived α1D/1A antagonist 1- benzyl-N-(3-(4-(2-methoxyphenyl) piperazine-1-yl) propyl)-1H-indole-2- carboxamide (HJZ-12) has been reported from our laboratory, which exhibits high subtype-selectivity to both α_1D_- and α_1A_- AR (47.9- and 19.1- fold, respectively) with respect to a1B-AR *in vitro*. However, no further study was conducted. In the present study, a pharmacological evaluation of HJZ-12 in BPH was performed on an estrogen/androgen-induced rat BPH model and human BPH-1 cell line. *In vivo*, HJZ-12 exhibited better performance than NAF in preventing the progression of rat prostatic hyperplasia by not only decreasing prostate weight and proliferation (similar to NAF) but also, shrinking prostate volume and inducing prostate apoptosis (different from NAF). *In vitro*, HJZ-12 exhibited significant cell viability inhibition and apoptotic induction in BPH-1 cell line, without presenting cell anti-proliferation properties. Intriguingly, the role of HJZ-12 on cell viability and apoptosis was an α1-independent action. Furthermore, RNA-Seq analysis was applied to screen out six anti-apoptotic genes (Bcl-3, B-lymphoma Mo-MLV insertion region 1 [Bmi-1], ITGA2, FGFR3, RRS1, and SGK1). Amongst them, Bmi-1 was involved in the apoptotic induction of HJZ-12 in BPH-1. Overall, HJZ-12 played a remarkable role in preventing the progression of prostatic hyperplasia through α1-independent apoptotic induction, indicating that it will be a multi-target effective candidate for BPH treatment.

## Introduction

Prostatic enlargement secondary to benign prostatic hyperplasia (BPH) resulting in lower urinary tract symptoms (LUTS) is a common disease with prevalence increasing with age (Parsons, 2010; Aaron et al., 2016). BPH is a multifactorial disease that is not yet fully understood. It was assumed that the development of BPH has both static and dynamic components relating to the prostate. The static component is related to increased prostatic tissue mass, whereby progressive proliferation leads to increased prostatic size and consequent bladder outlet obstruction (BOO). BPH is characterized by a proliferation of both stromal and epithelial cells of the prostate in the transitional zone surrounding the urethra (Strand et al., 2017). The dynamic component is related to variations in smooth muscle tone in the fibromuscular stroma, prostatic capsule, and bladder neck (Lepor, 2007; Mcvary et al., 2011; Chughtai et al., 2016). Therefore, α_1_-adrenergic receptor (α_1_-AR) antagonists are considered for the first line treatment in men with small prostate and 5α-reductase inhibitors are recommended in men with large symptomatic prostate (Van Asseldonk et al., 2015; Lim, 2017).

The therapeutic benefit of α_1_-AR antagonists in the medical treatment of BPH has been confirmed; they act directly on α_1_-AR, which is present in prostate smooth muscle. However, recent studies indicated that quinazoline based on α_1_-antagonists doxazosin inhibits proliferation of human vascular smooth muscle cells, which is unrelated to its capacity to occupy α_1_-AR (Hu et al., 1998). Clinical data also indicated that doxazosin and terazosin induced prostate apoptosis without affecting the proliferative capacity of prostate cells in patients with BPH (Kyprianou et al., 1998; Chon et al., 1999). Terazosin and doxazosin could also suppress prostate tumor via apoptotic induction by increasing TGF-β1 protein expression, and they are independent of the property of α_1_-AR antagonism (Kyprianou and Benning, 2000; Benning and Kyprianou, 2002; Partin et al., 2003). Thus, rapid evidences have implicated the apoptosis-mediated prostate stromal regression as an additional molecular mechanism underlying the long-term therapeutic effect of these drugs in BPH, rather than simply a pure smooth muscle relaxation of the prostate (Kyprianou, 2003).

Naftopidil (NAF, Supplementary Figure S1) is an arylpiperazine-based α_1D/1A_-AR antagonist used for treating BPH/LUTS in clinic. Unlike terazosin or doxazosin, NAF mainly blocks α_1D_ subtype and has 17- and three-fold higher potency for α_1D_-AR than for the α_1B_ and α_1A_-ARs, respectively (Takei et al., 1999). It was reported that NAF exhibits growth inhibition effects on human prostate cancer cell lines via G1 cell cycle arrest, indicating its potential benefit in the chemoprevention of prostate cancer (Kanda et al., 2008; Hori et al., 2011). Moreover, Kojima et al. has demonstrated that NAF has significant antiproliferation activity without apoptosis in BPH patients. NAF may prevent further development of prostate growth although it may not reduce prostate volume in patients with BPH (Kojima et al., 2009). α_1D_-AR may play a significant role on prostate growth in BPH development; thus, the inhibitory effect of prostate growth by NAF may probably involve an α_1D_-AR-dependent mechanism (Xin et al., 1997; Shibata et al., 2003).

The authors of the present study have been involved in the molecular optimization of NAF, pharmacological assays, and structural activity relationship (SAR) research for a long time. In a previous study, piperazine-derived α_1_ antagonist 1-benzyl-N-(3-(4-(2-methoxyphenyl) piperazin-1-yl) propyl) -1H-indole-2-carboxamide (HJZ-12) exhibited high subtype selectivity on α_1D_ and α_1A_-ARs (47.9- and 19.1- fold, respectively, Table 1) with respect to *a*_1B_; these results are better than those obtained in NAF and tamsulosin (Huang et al., 2015). In the present study, HJZ-12 was hypothesized to suppresses prostate cell growth in BPH by inducing apoptosis or affecting proliferation. The inhibitory action of HJZ-12 on prostate growth in rat BPH model and human BPH epithelial cell line (BPH-1) *in vitro* was investigated to test this hypothesis. The findings documented the ability of HJZ-12 to induce apoptosis by downregulating Bmi-1 protein, potentially via α_1_-AR-independent action. These results may have considerable therapeutic implications in identifying HJZ-12 as a potential multi-target candidate in BPH/LUTS treatment.

**TABLE 1:** ^a^Antagonist affinities of compounds HJZ-12, expressed as pA2, at α1A-, α1B-, and α1D-AR on isolated rat tissues.

Comp	p*A*_2_			Affinity ratio		
	α_1A_ prostatic vas deferens	α_1B_ spleen	α_1D_ thoracic aorta	α_1D_/α_1B_	α_1A_/α_1B_	α_1D_/α_1A_
HJZ-12	8.13 ± 0.01	6.85 ± 0.01	8.56 ± 0.06	47.9	19.1	2.6
NAF	7.48 ± 0.30	6.75 ± 0.01	7.93 ± 0.13	15.1	5.4	2.8
Tamsulosin	9.46 ± 0.13	9.30 ± 0.08	10.00 ± 0.10	5.0	1.4	3.5
Terazosin	7.90 ± 0.15	8.59 ± 0.08	8.83 ± 0.17	1.7	0.2	8.5

^a^ pA2 values ± SEM (n = 5–8) from previous data (Huang et al., 2015).

## Materials and Methods

### Chemicals and Reagents

Testosterone propionate, estradiol benzoate and olive oil were purchased from Aladdin Co., Ltd. (Shanghai, China). NAF (>99%) and HJZ-12 (>95%) were synthesized in according with the previous methods (Huang et al., 2015). Pierce BCA Protein Assay Kit was purchased from Thermo Fisher Scientific (Waltham, MA, United States). Anti-α-smooth muscle actin (α-SMA, 1/400) was purchased from Sigma-Aldrich (MO, United States). Anti-β-actin antibody (1/5000), Bcl-3 polyclonal antibody (1/500) and RRS1 polyclonal antibody (1/500) were purchased from Bioworld Technology (Inc., United States). Rabbit monoclonal anti-proliferating cell nuclear antigen (PCNA, 1/1000), and anti-FGFR3 antibody (1/1000) were purchased from Abcam (Cambridge, MA, United States). Anti-Bmi-1 antibody (1/1000) and anti-cleaved caspase 3 (1/1000) were purchased from Cell Signaling Technology (Beverly, MA, United States). All reagents were prepared fresh before use.

### *In Vivo* Study in Rat BPH Model Induced by E/T

Sprague–Dawley (SD) rats were obtained from Southern Medical University Laboratory Animal Center (Guangzhou, China). Prior to drug administration, the rats were handled and treated in accordance with the guidelines of the European Union Directive 2010/63/EU. The animal use and care protocols were reviewed and approved by the Ethics Committee of Guangzhou Medical University (approval number GY 2018-038).

The male SD rats (180–200 g) were divided into six experimental groups, including a model control, a sham-operated group, three HJZ-12 (low, middle and high doses) groups, and a positive control (NAF treatment group). The rats were anesthetized with intraperitoneally injecting 3% sodium pentobarbital. The testes of animal were removed in all the group except in the sham-operated group. After that gentamycin (0.1 ml/d) was given to the rat for 1 week. Then, E/T at a ratio of 1:100 (10/1000 μg) (Zhou et al., 2009; Huang et al., 2016) was applied daily in the castrated rats by subcutaneous injecting 0.1 ml of olive oil for 4 weeks, and the sham-operated group is only treated by olive oil without hormones.

### Analysis of Wet Weight Index and Volume Index of Rat Prostate

After the rats were sacrificed, the whole prostate of rat (anterior, ventral, lateral and dorsal lobes) was dissected out and weighed immediately, and the prostate volume was subsequently measured. The wet weight index (mg/g) of the prostate and bladder and the volume index (cm^3^/100 g) of the prostate were calculated as follows: wet weight index = wet weight of the prostate or bladder (mg)/body weight (g); volume index = volume of the prostate (mg)/body weight (g) × 100. The ventral and lateral lobes of prostate were used for other experiments in this study.

### Histological and Immunohistochemical Studies

Hematoxylin and eosin (HE) staining was carried out according to the standard protocols. Morphologic changes, including the relative volume of acinus, stroma and epithelium in rat prostate, and the epithelial height of rat prostate, were quantitatively analyzed using an image analysis system (Zeiss AXio Imager Z2, Germany). The expression of α-smooth muscle actin (α-SMA) was detected by anti-α-SMA antibody (1/400; MO, United States). The slide area was divided into 4 × 4 squares, and 10 randomly selected fields (200 × magnification) were examined from each section with four sections analyzed per animal (Yang et al., 2010; Huang et al., 2016).

### Western Blot Analysis

The total protein of concentration of each tissue or cell sample was determined by the Pierce BCA Protein Assay Kit (Waltham, MA, United States). 12% SDS-polyacrylamide gel and polyvinylidene-difluoride (PVDF) membranes (Millipore, Burlington, MA, United States) was used, and the membrane was blocked for 1 h at room temperature with 5% non-fat milk. The primary antibodies was incubated overnight at 4°C, including rabbit monoclonal anti-PCNA, anti-FGFR3 antibody; anti-β-actin antibody (1/5000), Bcl-3 polyclonal antibody and RRS1 polyclonal antibody (1/500; Bioworld Technology, Inc., United States); and anti-Bmi-1 antibody and anti-cleaved caspase3 (1/1000; CST, Beverly, MA). Then, the HRP-conjugated anti-mouse or anti-rabbit IgG (1/5000; Sigma–Aldrich MO, United States) was incubated for 1 h at room temperature. The enhanced chemiluminescence (ECL) western blotting detection reagents (GE Healthcare Biosciences, Pittsburgh, PA, United States) was used to get the band. The band was analyzed by ImageJ software.

### Cell Line and Cell Culture

Benign human prostatic epithelial cell line (BPH-1), purchased from Guangzhou Huiyuan Pharmaceutical Technology (Guangzhou, China), were cultured in RPMI 1640 medium (Gibco, United States) containing 10% fetal bovine serum (FBS) in a humidified incubator at 37°C and 5% CO_2_. Human prostatic stromal myofibroblast cell line (WPMY-1), purchased from Shanghai Zhong Qiao Xin Zhou Biotechnology Technology (Shanghai, China), were cultured in DMEM (Gibco, United States) containing 10% FBS in a humidified incubator at 37°C and 5% CO_2_.

### Cell Viability Assay

BPH-1 or WPMY-1 cell lines were seeded as 1 × 10^4^ cells per well in 96-well plates, with 100 μL culture medium. After 24 h, the cells were treated with compounds (0–100 μM) or vehicle for 24 h or 48 h. CCK-8 reagent was applied according to instruction (Dojindo Molecular Technologies, Japan), and the 450 nm was used as the absorption wavelength in a 96-cell spectrophotometric plate reader. The concentration required to inhibit cell growth by 50% (IC_50_) was calculated from inhibition curves.

### Cell-Cycle Analysis

Cell-cycle analysis was performed using a fluorescent probe PI as previously described. The BPH-1 cells (3 × 10^5^/well) were seeded in six-well plates and then cultured for 24 h. They were treated with different concentrations of HJZ-12 (0, 5, 10, and 15 μM) for 24 h, fixed at 70% ethanol, and then stored at 4°C for subsequent cell-cycle analysis. The fixed cells were washed with phosphate-buffered saline (PBS), incubated with PBS containing 20 μg/ml of RNaseA and 0.3% NP-40 for 30 min at 37°C, and then stained with 50 μg/ml of PI for 30 min at 4°C in the dark. The PI fluorescence emitted from HJZ-12-treated cells was measured using an Epics XL cytometer (Beckman Coulter, Brea, CA, United States). Data were analyzed on MultiCycle AV software (Phoenix Flow Systems, SanDiego, CA, United States).

### Detection of Apoptosis via Flow Cytometry

The BPH-1 cells were seeded in six-well plates and then cultured for 24 h. The cells were treated with different concentrations of HJZ-12 (0, 5, 10, and 15 μM) for 24 h, collected, and then stained using an Alexa Fluor^®^ 488Annexin V/dead cell apoptosis kit in accordance with the manufacturer’s protocol. The green fluorescence from the Alexa Fluor^®^ 488 Annexin V and the red fluorescence from PI were detected using an Epics XL cytometer (*E*_x_ = 488 nm and *E*_m_ = 530 nm for Alexa Fluor^®^ 488 Annexin V and *E*_m_ = 575 nm for PI) (Huang et al., 2015). Data were quantitatively analyzed on EXPO32 ADC software (Beckman Coulter, Brea, CA, United States). The cell population in the lower left (Annexin V^−^/PI^−^), lower right (Annexin V^+^/PI^−^) and upper right (Annexin V^+^/PI^+^) quadrants represents live, early apoptotic and late apoptotic or dead cells, respectively.

### TUNEL Analysis

TUNEL assay was performed using the *in-situ* cell death detection kit from Roche in accordance with the manufacturer’s instructions. In brief, after the cells were fixed with 4% paraformaldehyde and permeabilized in 0.1% Triton X-100, TUNEL assay was carried by incubating the fixed cells with a TUNEL reagent containing TMR red-labelled nucleotides at 37 °C for 1 h. The samples were washed in 1 × PBST and mounting media containing DAPI. Fluorescent images were captured using a Zeiss fluorescence microscope at 10× magnification. The total number of DAPI-positive cells and total number of TUNEL-positive cells were counted from at least five images from each sample. Each experiment was repeated four times.

### RNA-Seq Assay

The BPH-1 cells were treated with HJZ-12 (15 μM) for 12 h. RNA was extracted using a TRIzol reagent (Life Technologies, Foster City, CA, United States). After the rRNA was removed, RNAs were fragmented and reverse transcribed using random primers. cDNAs were ligated with adaptors, amplified via PCR and then sequenced on an Illumina HiSeqTM 2500 sequence analyzer. Filtered sequences were aligned with HISAT2, and gene expression was analyzed on DEGseq software. The differentially expressed genes were subjected to Gene Ontology (GO) and KEGG pathway analysis and were used to create heatmaps on the heatmap package of R (Huang et al., 2019).

### Bmi-1 Small Interfering RNA (siRNA) and Control SiRNA Transfection

At a concentration of 5 nM, Bmi-1 siRNA and control siRNA (RiboBio Inc., Guangzhou, Guangdong, China) were transfected into BPH-1 cells. The BPH-1 cells were cultured at 37°C in a CO_2_ incubator until the cells were 50%–60% confluent. Subsequently, the cells were transfected with RNAiMAX (Thermo Fisher Scientific, Waltham, MA, United States) in accordance with the manufacturer’s instructions. The transfection reagent and Bmi-1-siRNA (60 nM) or control-siRNA were incubated with BPH-1 cells in Opti-MEM (Gibco, United States) for 10 h, and then the complete media were changed into RPMI 1640 medium (Gibco, United States) containing 10% fetal bovine serum (FBS) for 14 h. Subsequently, the BPH-1 cells were treated with HJZ-12 (15 μM) for 24 h. Protein was collected for Western blot.

### Quantitative Real-Time PCR

Total cellular RNA was extracted using a TRIzol reagent (Life Technologies, Foster City, CA, United States). Reverse transcription was carried out with PrimeScript RT Master Mix (Takara, Otsu, Shiga, Japan) in accordance with the manufacturer’s instructions. Then, qRT-PCR was performed with SYBR Premix Ex Taq (Takara). GAPDH was used as an internal control. The following primers were used: **Bmi-1**, forward: 5′-TGG ACT GAC AAA TGC TGG AGA-3′ and reverse: 5′-GAA GAT TGG TGG TGG TTA CCG CTG-3′; **Bcl-3**, forward: 5′-TTC CTC TGG TGA ACC TGC CTAC-3′ and reverse: 5′-CGT GTC TCC GTC CTC ATC TGC-3′; **RRS1**, forward: 5′-CCC TAC CGG ACA CCA GAG TAA-3′ and reverse 5′-CCG AAA AGG GGT TGA AAC TTCC-3′; **ITGA2**, forward: 5′-ACT GTT CAA GGA GGA GAC-3′ and reverse 5′-GGT CAA AGG CTT GTT TAGG-3′; **FGFR3**, forward: 5′-GGG CTT CTT CCT GTT CAT CC-3′ and reverse 5′-TGG ACT CCA GGG ACA CTG TTA-3′; **SGK1**, forward: 5′-GAT CTC CCA ACC TCA GGA GCC-3′ and reverse 5′-CTG GAA AGA GAA GTG AAG GCCC-3′; and **GAPDH**, forward 5′-GAA GGT CGG AGT CAA CGG ATT-3′ and reverse 5′-CGC TCC TGG AAG ATG GTG AT-3′.

### Statistical Analysis

All data were presented as means ± SD. All other data were analyzed using a one-way ANOVA coupled with Turkey’s multiple comparisons test. *p* < 0.05 was the minimal requirement for a statistically significant difference. The statistical significance in this study was set at ^*^ *p* < 0.05, ^**^ *p* < 0.01, and ^***^ *p* < 0.001. All statistical analyzes were conducted on GraphPad Prism 8.0.

## Results

### Effects of HJZ-12 on Wet Weight and Volume Indices of Rat Prostate

In the BPH model group, the values of wet weight, wet weight index, volume, and volume index of the prostate were significantly larger than those in the sham-operated group (Table 2, *p* < 0.001). As an α_1_-AR blocker, NAF (10.0 mg/kg) had moderate effects on inhibiting both prostate wet weight and wet weight index (*p* < 0.01) compared with model group. However, no significant influence on prostate volume and volume index (*p* > 0.05) was observed, which was in accordance with the results of previous reports (Kojima et al., 2009; Huang et al., 2016; Liu et al., 2020).

**TABLE 2:** Effect of HJZ-12 treatment on wet weight and volume of the rat prostate.

Groups	Wet weight (mg)	Volume (cm^3^)	Wet weight index (mg/g)	Volume index (cm^3^/100 g)
Sham operated	902 ± 109.4^a^	0.98 ± 0.05^a^	2.79 ± 0.29^a^	0.30 ± 0.02^a^
Model control	2972 ± 85.21	2.57 ± 0.10	10.51 ± 0.35	0.91 ± 0.04
NAF (10.0 mg/kg)	2350 ± 76.68^b^	2.23 ± 0.04	8.08 ± 0.25^b^	0.75 ± 0.02
HJZ-12 (1.0 mg/kg)	2010 ± 119.8^a^	2.02 ± 0.11^c^	7.32 ± 0.44^a^	0.73 ± 0.04^c^
HJZ-12 (3.0 mg/kg)	1919 ± 159.4^a^	1.94 ± 0.18^b^	6.59 ± 0.45^a^	0.65 ± 0.05^b^
HJZ-12 (10.0 mg/kg)	1684 ± 87.85^c,d^	1.90 ± 0.16^b^	5.48 ± 0.37^c,d^	0.63 ± 0.05^b^

Values shown are the mean ± SD of 8 animals per group.

^a^ *p* < 0.001 compared with model control group.

^b^ *p* < 0.01 compared with model control group.

^c^ *p* < 0.05 compared with model control group.

^d^ *p* < 0.01 compared with NAF (10.0 mg/kg) group.

Compound HJZ-12 significantly decreased the wet weight index and volume index of rat prostate from low to high concentration (from 1.0 mg/kg to 10.0 mg/kg, *p* < 0.05) compared with model group. At an identical dose of 10.0 mg/kg, HJZ-12 administration showed significantly better inhibitory action on prostate wet weight and wet weight index than the NAF group (*p* < 0.01). Moreover, HJZ-12 showed inhibitory activity on rat hyperplastic prostate volume and volume index compared with model group (*p* < 0.05), even at a low dose (1.0 mg/kg, *p* < 0.05, relative to model group), and such performance was quite different from that of the pro-drug NAF.

### Effects of HJZ-12 on the Histomorphology of Hyperplastic Prostate

**FIGURE 1:**
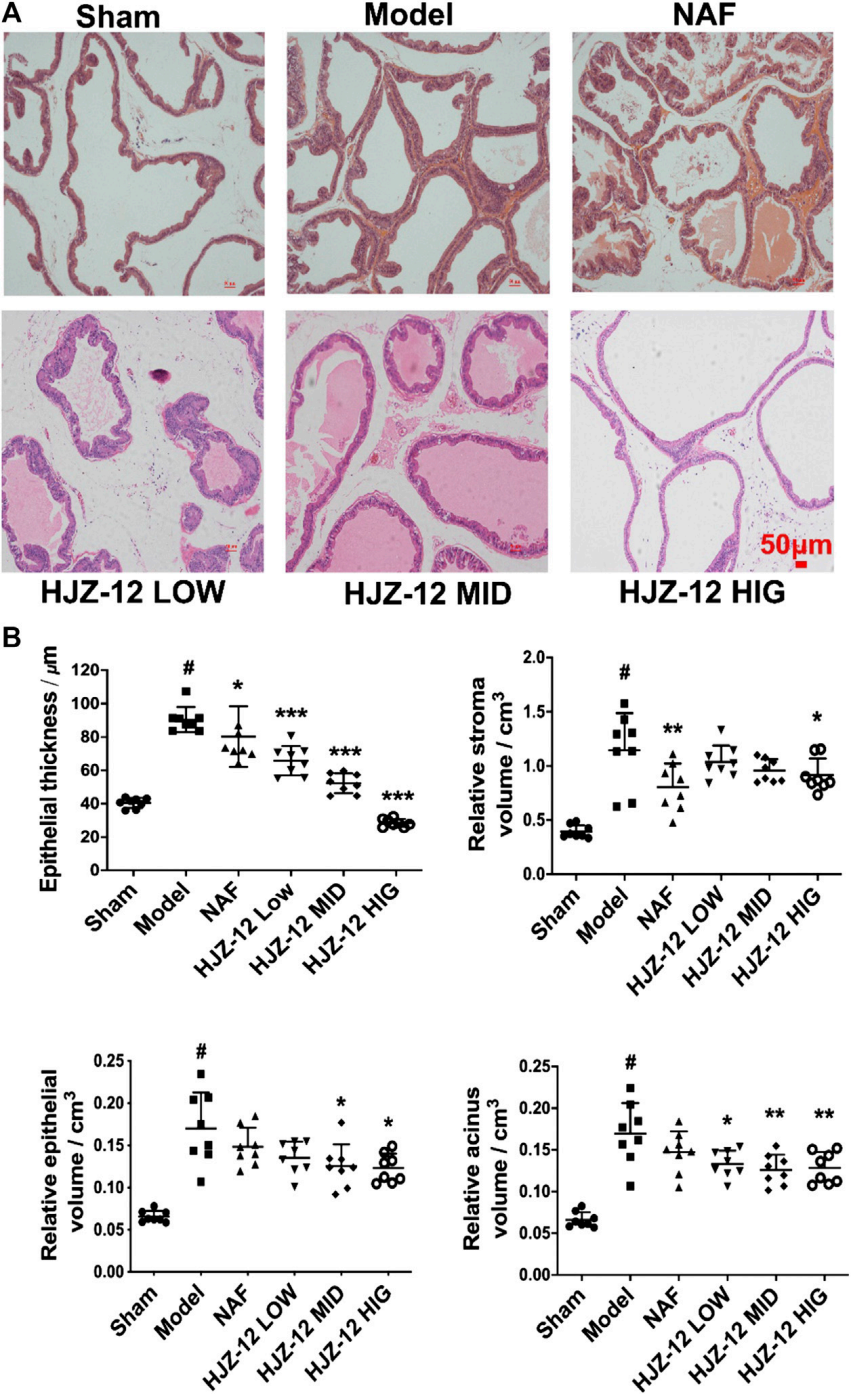
Histomorphological changes on rat prostate tissue. SD rats were orally administered with a sham (olive oil), model (E/T), NAF (10.0 mg/kg), HJZ-12 LOW (1.0 mg/kg), HJZ-12 MID (3.0 mg/kg) and HJZ-12 HIG (10.0 mg/kg) groups for 4 weeks. **(A)** Histomorphological images. Original magnification × 100. **(B)** Effect of HJZ-12 on epithelial thickness, relative stroma volume, relative epithelial volume and relative acinus volume of rat prostate. Columns, mean values; error bars, SD (n = 7–8). Significance was determined using one-way ANOVA coupled with Turkey’s multiple comparisons test. *p < 0.05, **p < 0.01, and ***p < 0.001 vs. the model control group. #*p* < 0.001 vs. the sham-operated group.

The lumens of prostate acini were normal in the sham-operated group, and the epithelial cells in the prostatic acini appeared cuboidal or columnar, and no obvious stromal hyperplasia was observed. In the model group, the prostatic acini were obviously dilated, and the glandular epithelial cells were markedly thickened. The most obvious change was observed in the groups of treated with high concentration of HJZ-12 (10.0 mg/kg), in which the prostatic acini returned to normal status, with clear arrangement and uniform size (Figure 1A).

Quantitative analysis (Figure 1B) showed that the epithelial thickness, relative stroma volume, relative epithelial volume, and relative acinus volume of the model group were significantly higher than those of the sham-operated group (*p* < 0.001). The NAF-treated group (NAF 10.0 mg/kg) showed no obvious decrease in the relative epithelial volume and acinus volume compared with the model group. The HJZ-12 (3.0 mg/kg and 10.0 mg/kg)-treated groups demonstrated reduced epithelial thickness (*p* < 0.001), relative epithelial volume (*p* < 0.05), and acinus volume (*p* < 0.01). Only the high dose of HJZ-12 inhibited the relative stroma volume (*p* < 0.05) and strongly reduced the epithelial thickness (*p* < 0.001).

### Effect of HJZ-12 on Prostate α-SMA and PCNA Expression

**FIGURE 2:**
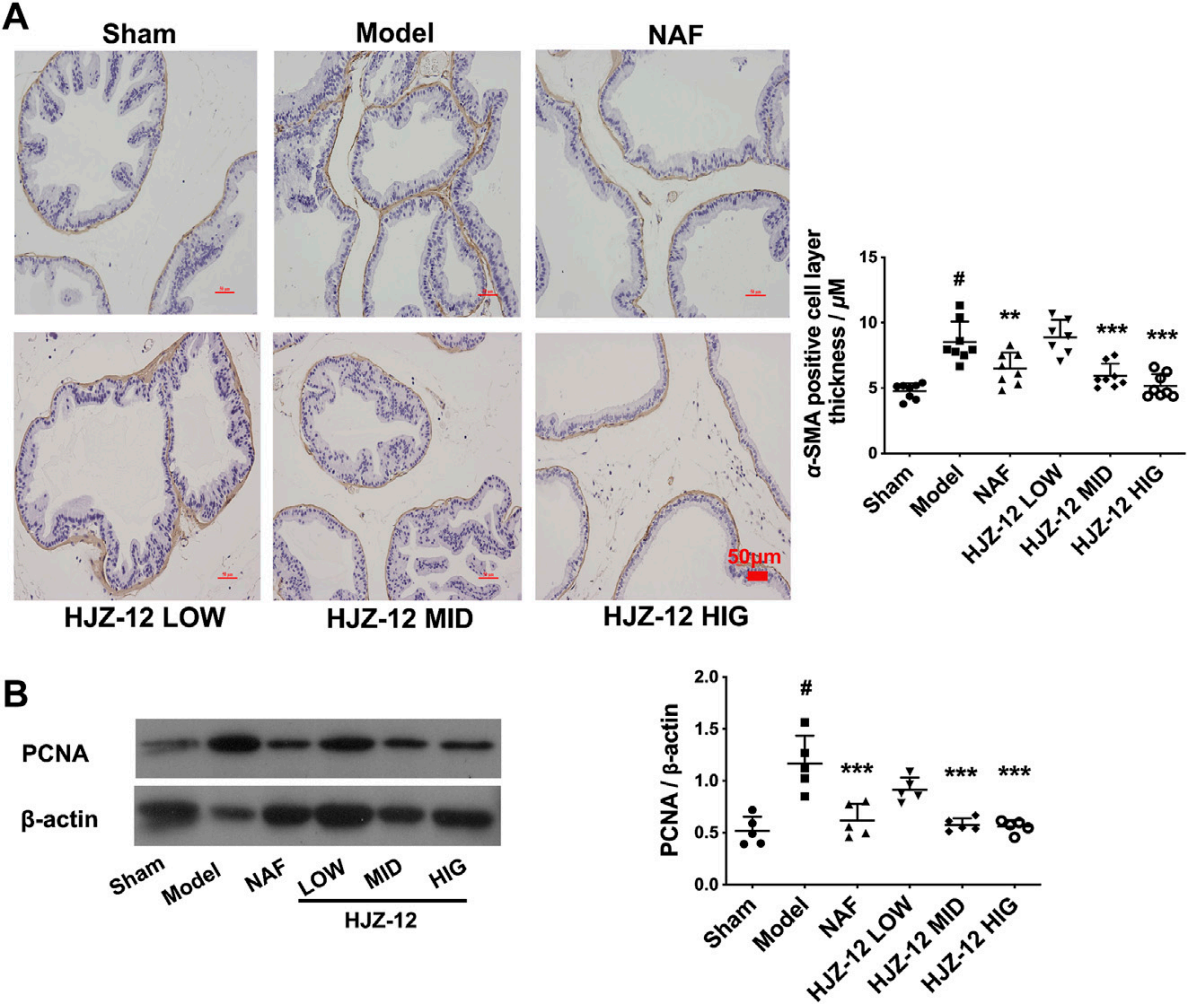
Effect of HJZ-12 on α-SMA and PCNA expression of rat prostate. SD rats were orally administered with a sham (olive oil), model (E/T), NAF (10.0 mg/kg), HJZ-12 LOW (1.0 mg/kg), HJZ-12 MID (3.0 mg/kg) and HJZ-12 HIG (10.0 mg/kg) groups for 4 weeks. **(A)** The α-SMA images in rat prostate, original magnification × 200; and quantitative analysis was determined by thickness (μM) of the layer surrounding the acinus. **(B)** PCNA expression in the prostate tissue determined using Western blot. Columns, mean values; error bars, SD (n = 5–8). Significance was determined using one-way ANOVA coupled with Turkey’s multiple comparisons test. **p < 0.01 and ***p < 0.001 vs. the model control group. #*p* < 0.01 vs. the sham-operated group.

In the BPH model, the acinus was surrounded with a SMA-positive smooth muscle layer compared with that in the sham-operated group (Figure 2A). Prostatic stromal cells are mainly composed of a large proportion of SMCs and fibroblasts, and a growing percentage of SMC is one of the major pathological changes in BPH. α-SMA is a microfilament protein with contractile properties that is widely distributed in stromal smooth muscle in BPH human prostate (Kyprianou et al., 1998; Quiles et al., 2010; Shimizu et al., 2015). The results of the present study showed that all the compound groups had reduced thickness of the SMA-positive layer, and the most significant reduction was obtained at a high dose of HJZ-12, indicating HJZ-12 could inhibit the extent of hyperplasia in stromal tissue in BPH to an extent.

In addition, PCNA was used as a proliferation marker in rat prostate (Bahey and El-Drieny, 2015). As shown in Figure 2B, PCNA protein expression was significantly increased in the prostate of the BPH model compared with that in the sham-operated group. Expectedly, NAF treatment obviously exhibited a decrease in PCNA expression. Low dose of HJZ-12 (1.0 mg/kg) showed no obvious effect on decreasing PCNA expression, however, middle and high doses of HJZ-12 markedly downregulated the PCNA protein expression (*p* < 0.001).

### Effect of HJZ-12 on Prostate Apoptosis

**FIGURE 3:**
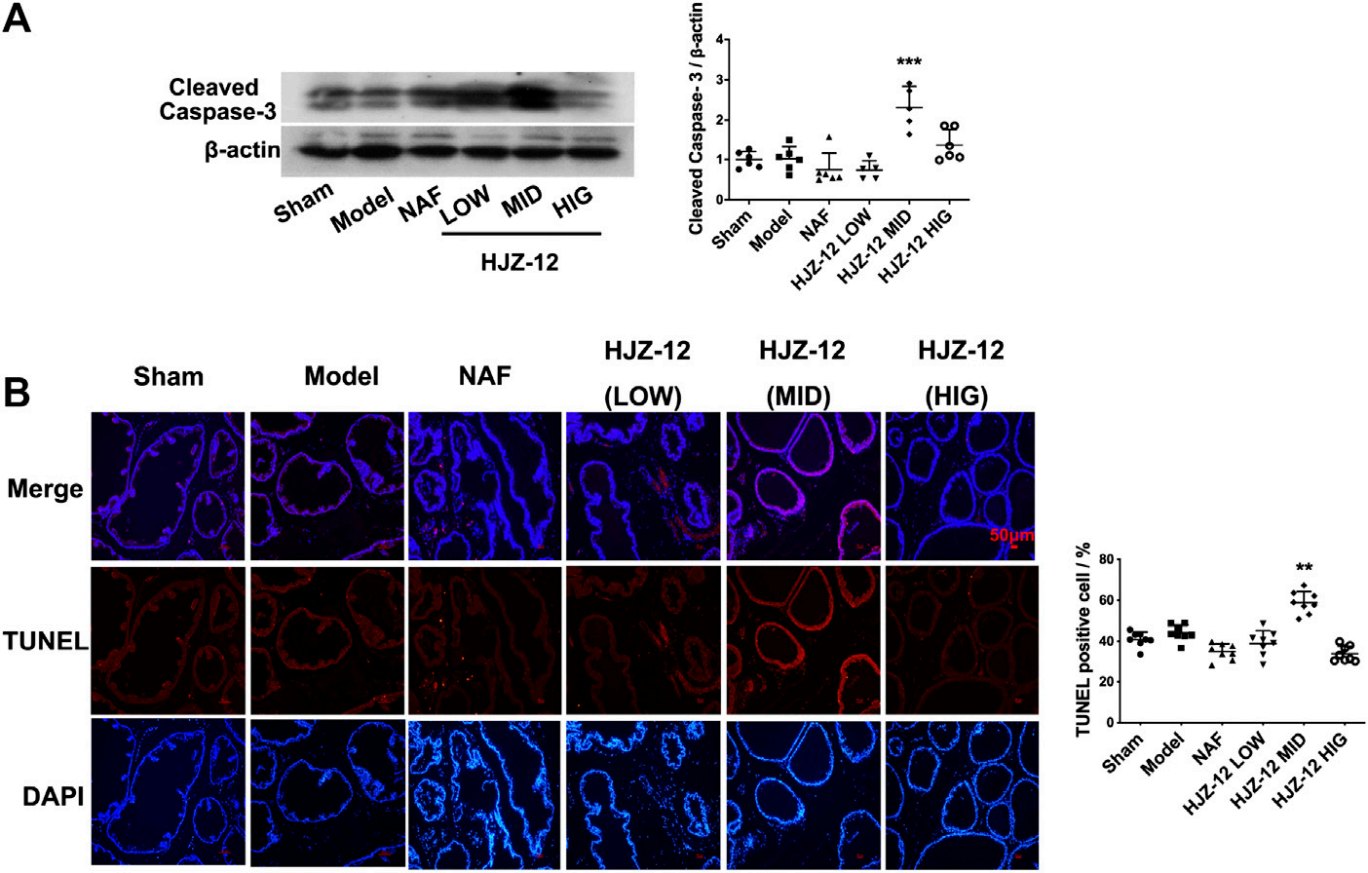
Effect of HJZ-12 on the apoptosis of rat prostate. **(A)** Protein lysates were collected from rat prostate treated with NAF (10.0 mg/kg), HJZ-12 LOW (1.0 mg/kg), HJZ-12 MID (3.0 mg/kg) and HJZ-12 HIG (10.0 mg/kg). Western blot and quantitative analysis were used to assess cleaved caspase-3 protein expression. **(B)** TUNEL assay was carried out to detect the apoptotic cells and TUNEL-positive cells were captured on a fluorescence microscope (100×). Columns, mean values; error bars, SD (n ≥ 5). Significance was determined using one-way ANOVA coupled with Turkey’s multiple comparisons test. **p < 0.01 and ***p < 0.001 vs. the model control group.

The effect of HJZ-12 on prostate apoptosis was subsequently tested. As shown in Figure 3A, a middle dose (3.0 mg/kg) of HJZ-12 markedly upregulated the cleaved caspase-3 protein level compared with the model control group (*p* < 0.001). Nevertheless, NAF and the other doses of HJZ-12 showed no effect on apoptosis. Next, apoptotic cells were measured using TUNEL assay. The middle dose (3.0 mg/kg) of HJZ-12 also significantly increased the TUNEL-positive cells in prostate tissue (*p* < 0.01, Figure 3B), which was consistent with the results of cleaved caspase-3. These results suggested that HJZ-12 rather than NAF showed a potent induction of prostate apoptosis. Thus, HJZ-12 reduced hyperplasia symptom on BPH, as indicated by the anti-proliferation and pro-apoptotic actions *in vivo*.

### Effect of HJZ-12 on BPH-1 and WPMY-1 Cell Viability

As shown in Table 3, NAF displayed no considerable cell viability inhibition on both BPH-1 in 24 h (IC_50_ > 50 μM). NAF also showed moderate inhibition of cell viability, with IC_50_ values of 40.33 ± 3.21 μM, in 48 h. By contrast, HJZ-12 caused 50% inhibition in 24 and 48 h at concentrations of 23.96 ± 1.05 and 14.76 ± 2.35 μM, respectively. Cell viability results on WPMY-1 cell line exhibited weaker performance compared with that on BPH-1 cells: IC_50_ values of HJZ-12 in 24 and 48 h were 39.13 ± 0.95 and 23.41 ± 1.21 μM, respectively.

**TABLE 3:** *In vitro* antiviability (IC_50_, μM)^a^ of HJZ-12 and NAF in BPH-1 and WPMY-1 cell lines.

	BPH-1		WPMY-1	
	24 h	48 h	24 h	48 h
HJZ-12	23.96 ± 1.05	14.76 ± 2.35	39.13 ± 0.95	23.41 ± 1.21
Naftopidil (NAF)	>50	40.33 ± 3.21	>50	48.59 ± 1.08

^a^ The cell viability inhibition of BPH-1 cell after treatment with different concentrations for 24 and 48 h, measured by CCK8 cell viability assay. The data expressed as inhibition percentage of control, are reported as IC_50_ values (n = 5), which represent the concentration (μM) of test compounds required to inhibit 50% of cell growth.

### Effect of HJZ-12 on Cell-Cycle and Cell Apoptosis in BPH-1 Cells

**FIGURE 4:**
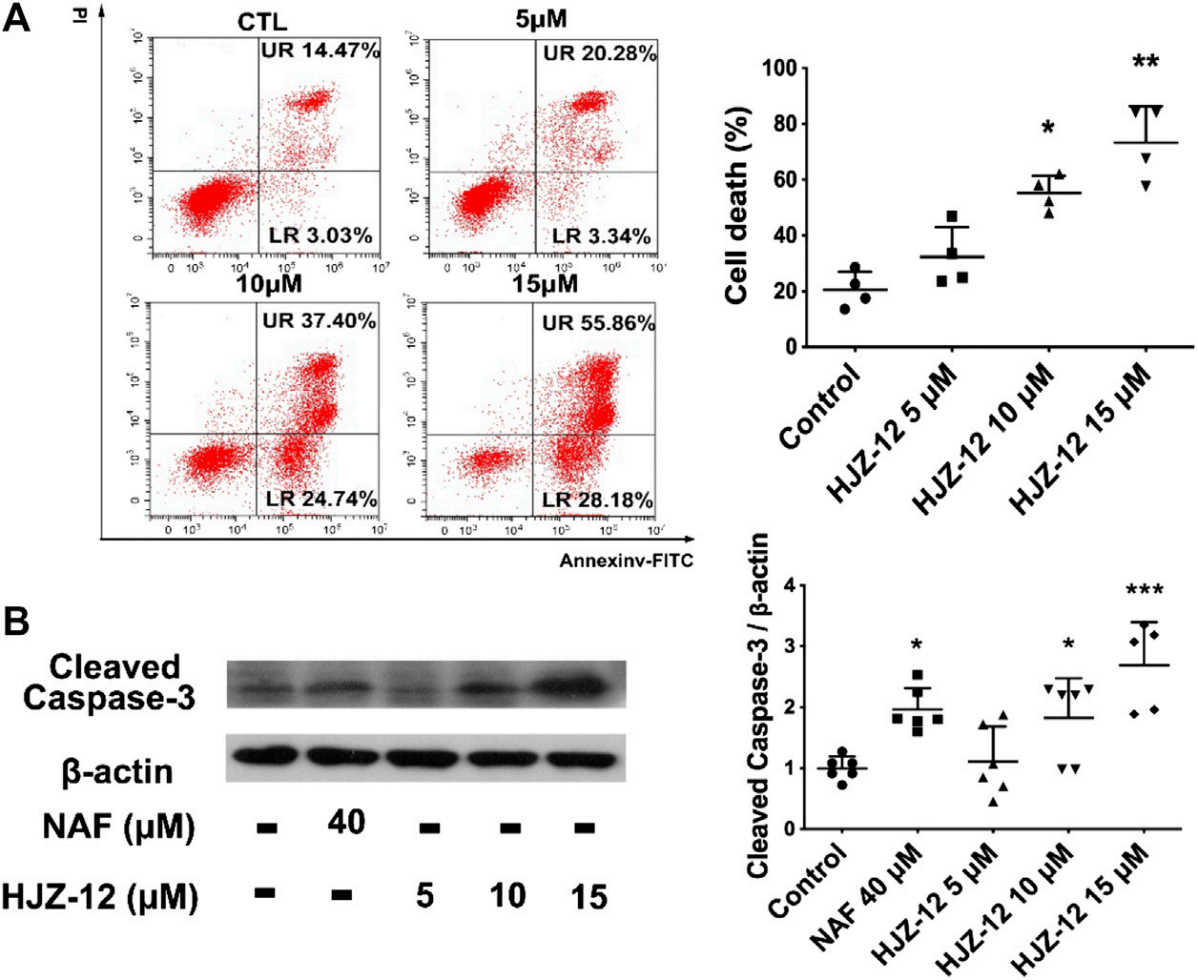
Apoptotic induction by HJZ-12 in BPH-1 cells. **(A)** BPH-1 cells treated with HJZ-12 (0, 5, 10, and 15 μM) for 24 h, were collected. Flow cytometry analysis with annexin V-FITC/PI staining was used to calculate the apoptotic cells. Representative images and cell death population analysis from three independent replicates are shown. **(B)** Protein lysates were collected from BPH-1 cells treated with HJZ-12 (0, 5, 10, and 15 μM) and NAF (40 μM) for 24 h. Western blot assay and quantitative analysis were used to assess cleaved caspase-3 protein expression. Columns, mean values; error bars, SD (n ≥ 4). Significance was determined using one-way ANOVA coupled with Turkey’s multiple comparisons test. *p < 0.05, **p < 0.01, and ***p < 0.001 vs. the control group.

As shown in Figure 4, HJZ-12 induced a notable apoptosis on the BPH-1 cell lines, however, without affecting the cell-cycle (Supplementary Figure S2). Flow cytometry assay showed that HJZ-12 increased the percentage of apoptotic cells in a dose-dependent manner in BPH-1 cells (Figure 4A), approximately by 23%, 62%, and 84% at 5, 10, and 15 μM, respectively. In addition, HJZ-12 dose-dependently induced caspase-3 activation (Figure 4B). HJZ-12 induced apoptosis rather than anti-proliferation in the BPH-1 cells.

### Effects of HJZ-12 on Norepinephrine- or Phenoxybenzamine-Treated BPH-1 Cells

**FIGURE 5:**
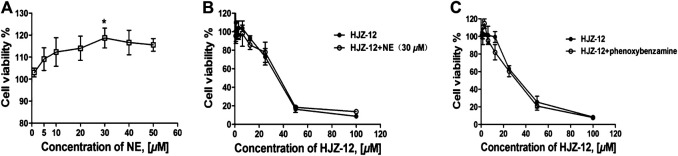
Effects of HJZ-12 on NE- or phenoxybenzamine-treated BPH-1 cells. **(A)** NE stimulated BPH-1 cell proliferation at different doses for 24 h. **(B)** Expression of the cell viability on BPH-1 cells treated with different doses of HJZ-12, alone or in combination with 30 μM NE was evaluated using CCK8 assays. **(C)** Expression of the cell viability on BPH-1 cells treated with different doses of HJZ-12, alone or in combination with 1 μM phenoxybenzamine, was evaluated using CCK8 assays. Columns, mean values; error bars, SD (n ≥ 4). Significance was determined using one-way ANOVA coupled with Turkey’s multiple comparisons test. *p < 0.05 vs. the control group.

Evidence indicated that activation of α_1_-ARs by catecholamines generally enhances growth-related gene expression and cell growth (Michelotti et al., 2000; Kojima et al., 2009). In the present study, the effect of HJZ-12 on norepinephrine (NE)- stimulated cell proliferation in BPH-1 cells was measured to determine whether or not the role of HJZ-12 on BPH-1 is associated with its antagonistic activity against α_1_-ARs. The endogenous agonist NE (0–50 μM) increased the cell viability from 0 to 30 μM and decreased upon 30 μM. At 30 μM, NE increased the cell viability by 15.2% (Figure 5A, *p* < 0.05). As shown in Figure 5B, treatment with NE (30 μM) did not change the viability inhibition of HJZ-12 in the BPH-1 cells (*p* > 0.05), suggesting an α_1_-adrenoceptor-independent effect of HJZ-12 on BPH-1 cell viability.

The catecholamine neurotransmitter NE binds to α_1_-ARs located on the cell membrane and activates phospholipase, generating a second messenger that ultimately results in smooth muscle contraction (Mcvary et al., 1998; Shibata et al., 2003). In order to confirm that whether the apoptotic effects of HJZ-12 were α_1_-AR mediated phenomenon, we subsequently tested the ability of irreversible of α_1_-AR inhibitor, phenxoybenzamine, to interfere with the antigrowth action of HJZ-12 (Hu et al., 1995; Kyprianou and Benning, 2000). As shown in Figure 5C, exposure to phenoxybenzamine (1 μM) prior to treatment did not inhibit the apoptotic effect of HJZ-12 against BPH-1. All these data indicated that the anti-growth effect of HJZ-12 on BPH-1 cells was mediated by α_1_ *-*adrenoceptor*-*independent mechanism.

### Bmi-1 Downregulation in HJZ-12-Induced BPH-1 Cell Apoptosis

**FIGURE 6:**
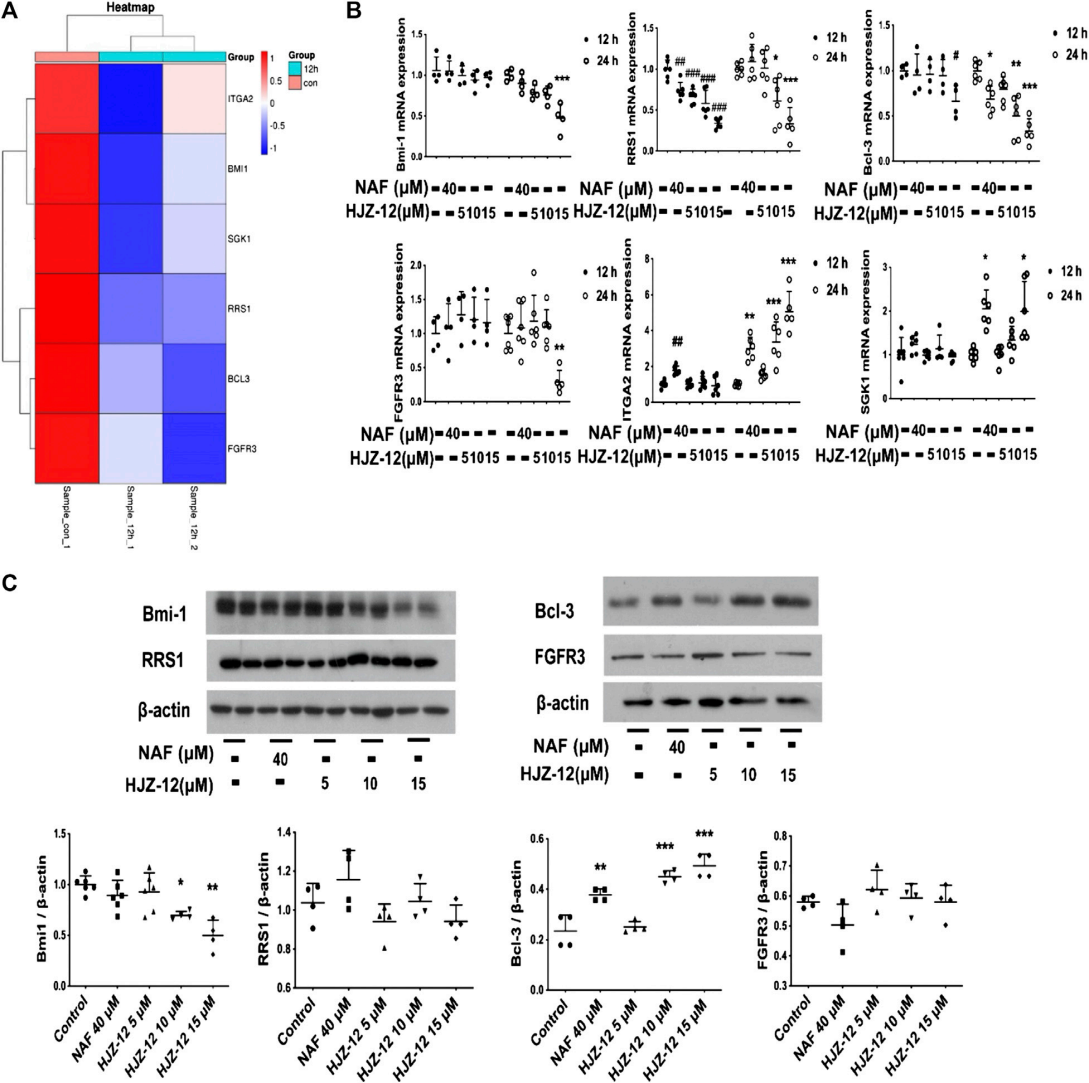
Screening out downregulation of Bmi-1 under HJZ-12 treatment. **(A)** Heatmap of RNA-seq data in BPH-1 cells treated with HJZ-12 (15 μM) for 12 h. **(B)** qPCR assay of six genes in BPH-1 cell during HJZ-12 (0, 5, 10, and 15 μM) and NAF (40 μM) treatments for 12 and 24 h #*p* < 0.05, ##*p* < 0.01, and ###*p* < 0.001 vs. the control group of 12 h; *p < 0.05, **p < 0.01, and ***p < 0.001 vs. the control group of 24 h. **(C)** Protein lysates were collected from BPH-1 cells treated with HJZ-12 (0, 5, 10, and 15 μM) and NAF (40 μM) for 24 h. Western blot assay and quantitative analysis were used to assess four proteins expression levels. *p < 0.05 and **p < 0.01 vs. the control group. Columns, mean values; error bars, SD (n ≥ 4). Significance was determined using one-way ANOVA coupled with Turkey’s multiple comparisons test.

To further investigate the mechanism underlying HJZ-12’s apoptotic induction, the BPH-1 cells were treated with HJZ-12 (15 μM) for 12 h, and the total RNA was extracted for high throughput sequencing (RNA-Seq). Downregulated sequenced-genes were screened using the following three limiting conditions, as follows: 1) fold change > 1.5, 2) gene expression is between 1 and 30 and 3) genes are closely related to anti-apoptosis (from PubMed database). Then, six genes, namely, Bcl-3, Bmi-1, FGFR3, ITGA2, RRS1, and SGK1, up to the above standards were screened out (Figure 6A). The RNA-Seq results were then confirmed via RT-qPCR and Western blot. First, the mRNA levels of BPH-1 cells treated with HJZ-12 at different concentrations (0, 5, 10, and 15 μM) for 12 and 24 h were detected using RT-qPCR. The mRNA levels of four genes (Bmi-1, FGFR3, RRS1, and Bcl-3) were significantly decreased, whereas the other two mRNA levels (SGK1 and ITGA2) were significantly increased (Figure 6B). Then, the expression levels of Bmi-1, FGFR3, RRS1, and Bcl-3 proteins in BPH-1 cells were analyzed using Western blot for 24 h. Bmi-1 protein expression was significantly decreased by HJZ-12 (10 and 15 μM) in accordance with its mRNA level (Figure 6C, *p* < 0.05 vs. negative control).

**FIGURE 7:**
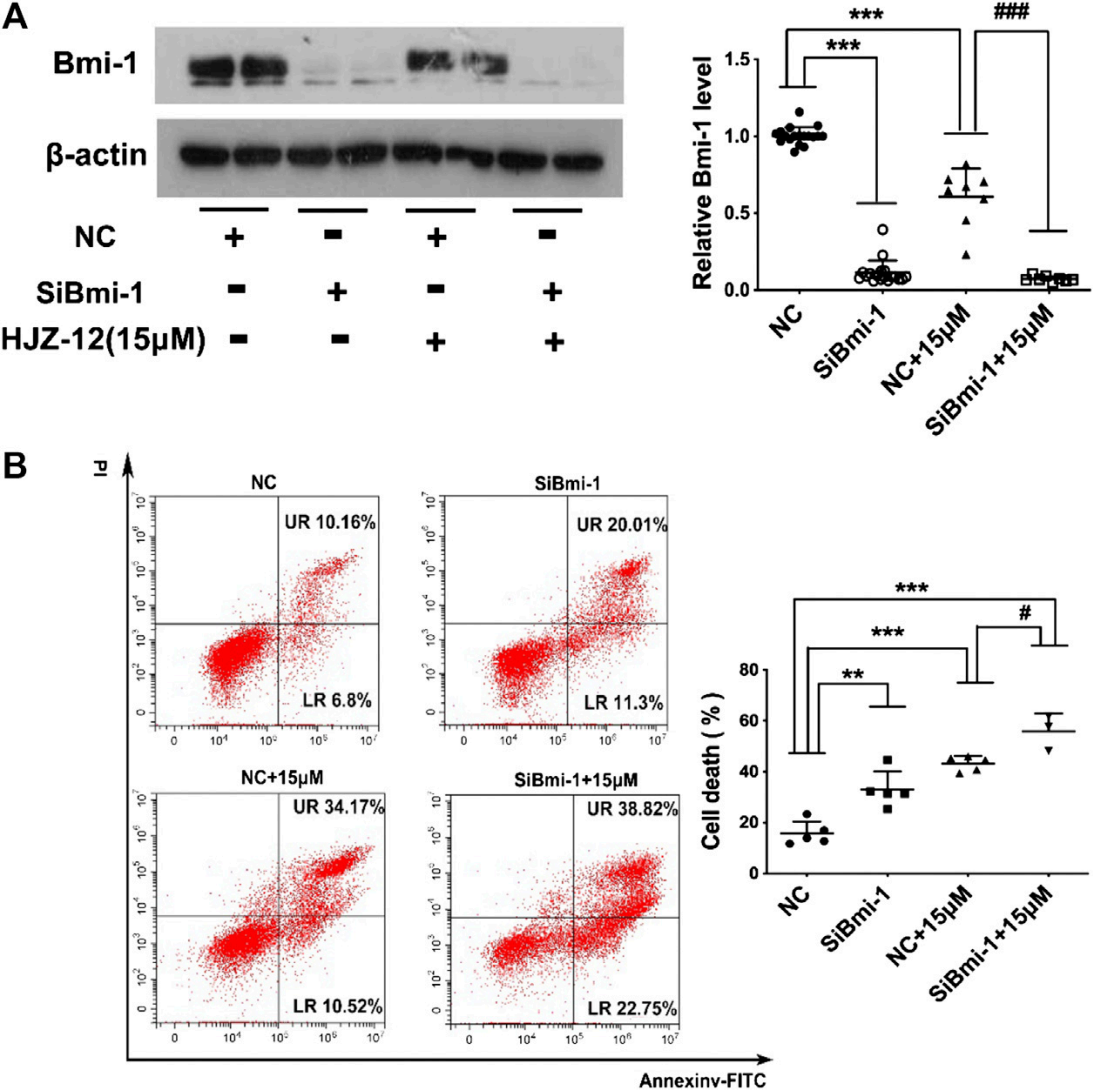
Bmi-1 mediates the apoptotic induction of HJZ-12 in BPH-1 cells. **(A)** Downregulation efficacy of Bmi-1 siRNA and HJZ-12 (15 μM) in BPH-1 cells as examined using Western blot, and quantitative analysis of the protein levels of Bmi-1 normalized to that of β-actin. ***p < 0.001 vs. the NC treatment group, ###*p* < 0.01 vs. the NC plus 15 μM HJZ-12 treatment group. **(B)** Effect of Bmi-1 downregulation on apoptosis of BPH-1 cells, as examined using flow cytometry analysis with Annexin V-FITC/PI staining was used to calculate the apoptotic cells. **p < 0.01 and ***p < 0.001 vs. the NC treatment group, #*p* < 0.05 vs. the NC plus 15 μM HJZ-12 treatment group. Columns, mean values; error bars, SD (n ≥ 5). Significance was determined using one-way ANOVA coupled with Turkey’s multiple comparisons test.

Next, Bmi-1 siRNA was applied to silence the Bmi-1 expression in BPH-1 cells, mimicking the Bmi-1 status in HJZ-12 treatment. As shown in Figure 7, we found that Bmi-1 protein expression was significantly reduced (*p* < 0.001), and the apoptotic rate was significantly increased compared with the NC group (*p* < 0.01), similar to the HJZ-12 groups. When Bmi-1 siRNA was provided in the presence of HJZ-12 (15 μM), Bmi-1 protein expression was further significantly reduced (*p* < 0.001) and the apoptotic rate was further increased, compared with the HJZ-12 (15 μM) group (*p* < 0.05). All the above results suggested that the downregulation of Bmi-1 protein involved in HJZ-12-induced BPH-1 apoptosis.

## Discussion

Combined α_1A_/α_1D_-selective antagonists maybe necessary for an optimal clinical benefit, as the presence of α_1D_- ARs in human bladder and spinal cord may play a role in the pathophysiology of prostatic disease (Schwinn and Michelotti, 2000; Unnikrishnan et al., 2017). Some recent studies have also demonstrated that α_1_-AR antagonists may affect prostate pathophysiology by inducing apoptosis via mechanisms that transcend smooth muscle relaxation (Garrison and Kyprianou, 2006). However, the effect whereby α_1D/1A_-AR blocker HJZ-12 suppresses the progress of BPH and induces apoptosis is not well understood. In the present study, an *in vivo* anti-BPH study of compound HJZ-12 in estrogen/androgen-induced rat BPH model was performed. The results showed that HJZ-12 effectively inhibited the development of BPH, as indicated by the decreased prostatic index, and inhibited PCNA and α*-*SMA expression in the prostate tissue. In addition, HJZ-12 induced apoptosis and decreased the hyperplastic prostate volume *in vivo*, which differs from NAF. HJZ-12 also inhibited the cell growth of BPH-1 *in vitro* without affecting cell cycle but inducing apoptosis via downregulation of Bmi-1 protein.

*In vivo*, compound HJZ-12 performed better anti-BPH effect than NAF under the same dose administration (10.0 mg/kg) in the rat BPH model. HJZ-12 exhibited clearer arrangement and more uniform size than NAF according to the histomorphologic image (Figure 1A). Moreover, HJZ-12 treatment deeply restrained prostate growth by not only decreasing wet weight index, but also obviously shrinking volume index (Table 2). Glandular weight was used to assess the progression of BPH, because BPH involves epithelial and stromal hyperplasia of the prostate that results in the increase in the prostate weight (Suzuki et al., 1994). Prostate volume is also an important marker in BPH-development, as an increasing ratio of estrogen and androgen stimulates the prostate enlargement, which causes anatomical obstruction to urine outflow (Nieto et al., 2014). NAF was reported to inhibit prostate cell proliferation, which is mediated through its α_1_-AR antagonism, as α_1D_-subtype plays an significant role in prostate growth (Xin et al., 1997). However, NAF does not induce apoptosis *in vivo* and has no inhibitory effect on prostate volume both in the rat BPH model and patients with BPH (Kojima et al., 2009). Considering the different performances between HJZ-12 and NAF, the characteristics of apoptosis may be a potential mechanism of the shrinking prostate size for HJZ-12 therapy in BPH.

*In vitro*, the initial mechanism research of HJZ-12 proceeded upon human BPH-1 cell lines. HJZ-12 and NAF caused 50% inhibition of cell growth in 48 h at concentrations of 14.76 ± 2.35 μM and 40.33 ± 3.21 μM, respectively. Similar with doxazosin and terazosin (Kyprianou and Benning, 2000; Kyprianou, 2003), our observation that HJZ-12 exerted a similar antigrowth effect against human A549 lung cancer cells and MCF-7 breast cancer cells (Supplementary Table S1), implying the apoptotic action of HJZ-12 is a global effect rather than prostate-specific character. α_1_-ARs coupled through G proteins modulated diverse intracellular processes, including activation of vascular smooth muscle contraction and promotion of proliferative responses. Therefore, some possibilities to attribute the cellular effects of HJZ-12 to its already found α_1_-ARs antagonism existed. However, in the NE-stimulated models, NE did not inhibit the anti-growth effect of HJZ-12 on BPH-1; this action was in accordance with the observations that pre-treatment with phenoxybenzamine did not abrogate the cellular response to HJZ-12- induced apoptosis in BPH-1 cells, neither. These findings indicated the special α_1_-independent role of HJZ-12 against BPH-1 cells, as opposed to that of NAF. It was reported that the α_1_-AR subtypes play a direct role in regulating the cell cycle in several cell types. Moreover, this regulation may be subtype specific, with the α_1A_- and α_1D_-ARs mediating cell cycle arrest, whereas α_1B_-AR causes cell-cycle progression and induces transformation (Gonzalez-Cabrera et al., 2004). Given that the results of the present study showed that HJZ-12 did not affect BPH-1 cell cycle arrest, NE should not inhibit the anti-growth effect of HJZ-12 upon BPH-1.

RNA-Seq assay and additional restricted conditions followed by RT-qPCR and Western blot verification showed that Bmi-1 protein was involved in HJZ-12-induced apoptosis in BPH-1 cells. The oncogenic polycomb group protein Bmi-1 regulates both normal and cancer stem cells in multiple tissues. Bmi-1 expression is necessary for normal prostate tubule regeneration *in vivo*. Bmi-1 inhibition protects prostate cells from FGF-10-driven hyperplasia and slows the growth of prostate cancer (Li et al., 2010; Lukacs et al., 2010). Most reports focused on the role of Bmi-1 in the development of prostate cancer. However, to what extent does Bmi-1 affect prostatic hyperplasia is unknown. In the present study, HJZ-12 could considerably inhibit Bmi-1 expression in an α_1_-independent manner and induce apoptosis, which was similar to Bmi-1-siRNA. Moreover, Bmi-1 siRNA further reduced the Bmi-1 level and triggered apoptosis in the presence of HJZ-12, implying that downregulation of Bmi-1 protein via an α_1_-adrenoceptor-independent mechanism possibly mediated HJZ-12-induced apoptosis. However, identifying the mechanism on how HJZ-12 decreased Bmi-1 expression to induce apoptosis in BPH-1 cells requires additional experiments.

BPH is a multifactorial disease that most likely require multi-target strategies to improve therapeutic efficacy (Morphy et al., 2004; Lu et al., 2012). The therapeutic benefit of α_1_- ARs antagonists to relax prostate muscle tone has been confirmed but it is insufficient for patients with big hyperplastic prostate size in clinical (Mcvary et al., 2011; Van Asseldonk et al., 2015). For the management of BPH, the use of antagonists to simultaneously relax the prostate and slow prostate enlargement may be more effective than monotherapy solely targeting α_1A_- and α_1D_-ARs (Hieble, 2011). However, the improvement of LUTS/BPH mediated by the association of 5α*-*reductase inhibitors with α_1_-AR blockers as compared with monotherapy with α_1_-AR blockers is only clearly observed after long term therapy; some adverse effect of 5α*-*reductase inhibitors might reduce patient compliance to treatment (Nascimento-Viana et al., 2016). Thus, the present study reported that HJZ-12, which could concomitantly relax prostate muscle and shrink hyperplastic prostate size, may be a multi-target effective candidate for the treatment of BPH/LUTS and modify the course of the disease.

In conclusion, piperazine-derived compound HJZ-12 suppressed BPH progress and induced apoptosis through deregulation of signal transduction pathways, potentially involving Bmi-1 signaling. Targeting apoptosis in an attempt to control prostatic growth may be a potentially powerful therapeutic approach for the effective treatment of BPH. Studies of more *in vitro* prostatic cell models are in progress to investigate the mechanistic aspects of the apoptotic effect of HJZ-12 against BPH. Overall, our study provided the possibility of multi-target drug design for treating BPH. Superior and multi-targeted drugs administration for patients with BPH are hopeful to be used in clinical in the near future.

## Data Availability Statement

The datasets analyzed in this article have been deposited in the Sequence Read Archive (SRA) database of NCBI under accession code PRJNA675922.

## Ethics Statement

The animal study was reviewed and approved by the Ethics Committee of Guangzhou Medical University (approval number GY 2018-038).

## Author Contributions

QX, FH, JH, and WL designed the project, and wrote the manuscript. QL, RJ, KC, XZ, and LM conducted the experiments and performed data analysis. All authors contributed to the article and approved the submitted version.

## Funding

This work was supported by National Natural Science Foundation of China (21807017), PhD Start-up Fund of Natural Science Foundation of Guangdong Province (2017A030310458), Science and Technology Planning Project of Guangdong Province (2017A020215194) and Foundation for Distinguished Young Talents in Higher Education of Guangdong Province (2015KQNCX135).

## Conflict of Interest

The authors declare that the research was conducted in the absence of any commercial or financial relationships that could be construed as a potential conflict of interest.

## Abbreviations

HJZ-12, 1- benzyl-N-(3-(4-(2-methoxyphenyl) piperazine-1-yl) propyl) -1H-indole-2- carboxamide; BPH, benign prostatic hyperplasia; LUTS, lower urinary tract symptoms; BOO, bladder outlet obstruction; α1-AR, α1-adrenergic receptor; NAF, naftopidil; BPH-1, human benign hyperplastic prostatic epithelial cell line; α-SMA, α-smooth muscle actin; PCNA, proliferating cell nuclear antigen; SMC, smooth muscle cell; NE, norepinephrine; Bmi-1, B-lymphoma Mo-MLV insertion region 1.

## References

[B1] Aaron L. Franco O. E. Hayward S. W. (2016). Review of prostate anatomy and embryology and the etiology of benign prostatic hyperplasia. Urol. Clin. North. Am. 43, 279–288. 10.1016/j.ucl.2016.04.012 27476121 PMC4968575

[B2] Bahey N. G. El-Drieny E. A. E. (2015). Immunoelectron microscope localization of androgen receptors and proliferating cell nuclear antigen in the epithelial cells of albino rat ventral prostate. J. Microsc. Ultrastruct. 3, 75–81. 10.1016/j.jmau.2015.01.002 30023185 PMC6014191

[B3] Benning C. M. Kyprianou N. (2002). Quinazoline-derived alpha1-adrenoceptor antagonists induce prostate cancer cell apoptosis via an alpha1-adrenoceptor-independent action. Cancer Res. 62, 597–602. https://cancerres.aacrjournals.org/content/62/2/597. 11809715

[B4] Chon J. K. Borkowski A. Partin A. W. Isaacs J. T. Jacobs S. C. Kyprianou N. (1999). Alpha 1-adrenoceptor antagonists terazosin and doxazosin induce prostate apoptosis without affecting cell proliferation in patients with benign prostatic hyperplasia. J. Urol. 161, 2002–2008. 10.1016/s0022-5347(05)68873-8 10332490

[B5] Chughtai B. Forde J. C. Thomas D. D. Laor L. Hossack T. Woo H. H. (2016). Benign prostatic hyperplasia. Nat. Rev. Dis. Primers. 2, 16031 10.1038/nrdp.2016.31 27147135

[B6] Garrison J. B. Kyprianou N. (2006). Doxazosin induces apoptosis of benign and malignant prostate cells via a death receptor-mediated pathway. Cancer Res. 66, 464–472. 10.1158/0008-5472.CAN-05-2039 16397262 PMC1850148

[B7] Gonzalez-Cabrera P. J. Shi T. Yun J. Mccune D. F. Rorabaugh B. R. Perez D. M. (2004). Differential regulation of the cell cycle by alpha1-adrenergic receptor subtypes. Endocrinology. 145, 5157–5167. 10.1210/en.2004-0728 15297446

[B45] Hieble J. P. (2011). Animal models for benign prostatic hyperplasia. Handb. Exp. Pharmacol. 202, 69–79. 10.1007/978-3-642-16499-6-4 21290222

[B8] Hori Y. Ishii K. Kanda H. Iwamoto Y. Nishikawa K. Soga N. (2011). Naftopidil, a selective {alpha}1-adrenoceptor antagonist, suppresses human prostate tumor growth by altering interactions between tumor cells and stroma. Cancer Prev. Res. (Phila). 4, 87–96. 10.1158/1940-6207.CAPR-10-0189 21205739

[B9] Hu Z. W. Shi X. Y. Hoffman B. B. (1998). Doxazosin inhibits proliferation and migration of human vascular smooth-muscle cells independent of alpha1-adrenergic receptor antagonism. J. Cardiovasc. Pharmacol. 31, 833–839. 10.1097/00005344-199806000-00006 9641467

[B10] Hu Z. W. Shi X. Y. Okazaki M. Hoffman B. B. (1995). Angiotensin II induces transcription and expression of alpha 1-adrenergic receptors in vascular smooth muscle cells. Am. J. Physiol. 268, H1006–H1014. 10.1152/ajpheart.1995.268.3.H1006 7900855

[B11] Huang J. He F. Huang M. Liu X. Xiong Y. Huang Y. (2015). Novel naftopidil-related derivatives and their biological effects as alpha1-adrenoceptors antagonists and antiproliferative agents. Eur. J. Med. Chem. 96, 83–91. 10.1016/j.ejmech.2015.04.005 25874333

[B12] Huang J. J. Cai Y. Yi Y. Z. Huang M. Y. Zhu L. He F. (2016). Pharmaceutical evaluation of naftopidil enantiomers: rat functional assays *in vitro* and estrogen/androgen induced rat benign prostatic hyperplasia model *in vivo* . Eur. J. Pharmacol. 791, 473–481. 10.1016/j.ejphar.2016.09.009 27615445

[B13] Huang Z. Zhang Y. Li H. Zhou Y. Zhang Q. Chen R. (2019). Vitamin D promotes the cisplatin sensitivity of oral squamous cell carcinoma by inhibiting LCN2-modulated NF-κB pathway activation through RPS3. Cell Death Dis. 10, 936–949. 10.1038/s41419-019-2177-x 31819048 PMC6901542

[B14] Kanda H. Ishii K. Ogura Y. Imamura T. Kanai M. Arima K. (2008). Naftopidil, a selective alpha-1 adrenoceptor antagonist, inhibits growth of human prostate cancer cells by G1 cell cycle arrest. Int. J. Cancer. 122, 444–451. 10.1002/ijc.23095 17918159

[B15] Kojima Y. Sasaki S. Oda N. Koshimizu T. A. Hayashi Y. Kiniwa M. (2009). Prostate growth inhibition by subtype-selective alpha(1)-adrenoceptor antagonist naftopidil in benign prostatic hyperplasia. Prostate. 69, 1521–1528. 10.1002/pros.21003 19544328

[B16] Kyprianou N. (2003). Doxazosin and terazosin suppress prostate growth by inducing apoptosis: clinical significance. J. Urol. 169, 1520–1525. 10.1097/01.ju.0000033280.29453.72 12629407

[B17] Kyprianou N. Benning C. M. (2000). Suppression of human prostate cancer cell growth by alpha1-adrenoceptor antagonists doxazosin and terazosin via induction of apoptosis. Cancer Res. 60, 4550–4555. 10969806

[B18] Kyprianou N. Litvak J. P. Borkowski A. Alexander R. Jacobs S. C. (1998). Induction of prostate apoptosis by doxazosin in benign prostatic hyperplasia. J. Urol. 159, 1810–1815. 10.1016/S0022-5347(01)63162-8 9598465

[B19] Lepor H. (2007). Alpha blockers for the treatment of benign prostatic hyperplasia. Rev. Urol. 9, 181–190. 10.1016/j.ucl.2016.04.009 18231614 PMC2213889

[B20] Li J. Gong L. Y. Song L. B. Jiang L. L. Liu L. P. Wu J. (2010). Oncoprotein Bmi-1 renders apoptotic resistance to glioma cells through activation of the IKK-nuclear factor-kappaB Pathway. Am. J. Pathol. 176, 699–709. 10.2353/ajpath.2010.090502 20035051 PMC2808077

[B21] Lim K. B. (2017). Epidemiology of clinical benign prostatic hyperplasia. Asian J. Urol. 4, 148–151. 10.1016/j.ajur.2017.06.004 29264223 PMC5717991

[B22] Liu Q. M. Xiao Q. Zhu X. Chen K. F. Liu X. W. Jiang R. C. (2020). Inhibitory effect of α1D/1A antagonist 2- (1H-indol-3-yl)-N- [3- (4- (2-methoxyphenyl) piperazinyl) propyl] acetamide on estrogen/androgen-induced rat benign prostatic hyperplasia model *in vivo* . Eur. J. Pharmacol. 870, 172817–172822. 10.1016/j.ejphar.2019.172817 31756334

[B23] Lu J. J. Pan W. Hu Y. J. Wang Y. T. (2012). Multi-target drugs: the trend of drug research and development. PloS One. 7, e40262 10.1371/journal.pone.0040262 22768266 PMC3386979

[B24] Lukacs R. U. Memarzadeh S. Wu H. Witte O. N. (2010). Bmi-1 is a crucial regulator of prostate stem cell self-renewal and malignant transformation. Cell stem cell. 7, 682–693. 10.1016/j.stem.2010.11.013 21112563 PMC3019762

[B25] Mcvary K. T. Roehrborn C. G. Avins A. L. Barry M. J. Bruskewitz R. C. Donnell R. F. (2011). Update on AUA guideline on the management of benign prostatic hyperplasia. J. Urol. 185, 1793–1803. 10.1016/j.juro.2011.01.074 21420124

[B26] Mcvary K. T. Mckenna K. E. Lee C. (1998). Prostate innervation. Prostate. 36, 2–13. 10.1002/(sici)1097-0045(1998)8+<2::aid-pros2>3.0.co;2-u 9690657

[B27] Michelotti G. A. Price D. T. Schwinn D. A. (2000). Alpha 1-adrenergic receptor regulation: basic science and clinical implications. Pharmacol. Ther. 88, 281–309. 10.1016/s0163-7258(00)00092-9 11337028

[B28] Morphy R. Kay C. Rankovic Z. (2004). From magic bullets to designed multiple ligands. Drug Discov. Today. 9, 641–651. 10.1016/s1359-6446(04)03163-0 15279847

[B29] Nascimento-Viana J. B. Carvalho A. R. Nasciutti L. E. Alcántara-Hernández R. Chagas-Silva F. (2016). New multi-target antagonists of α1A-, α1D-adrenoceptors and 5-HT1A receptors reduce human hyperplastic prostate cell growth and the increase of intraurethral pressure. J. Pharmacol. Exp. Ther. 356, 212–222. 10.1124/jpet.115.227066 26493747

[B30] Nieto C. M. Rider L. C. Cramer S. D. (2014). Influence of stromal-epithelial interactions on androgen action. Endocr. Relat. Cancer. 21, T147–T160. 10.1530/ERC-14-0138 24872510

[B31] Parsons J. K. (2010). Benign prostatic hyperplasia and male lower urinary tract symptoms: epidemiology and risk Factors. Curr. Bladder Dysfunct. Rep. 5, 212–218. 10.1007/s11884-010-0067-2 21475707 PMC3061630

[B32] Partin J. Anglin I. Kyprianou N. (2003). Quinazoline-based α1-adrenoceptor antagonists induce prostate cancer cell apoptosis via TGF-β signalling and IκBα induction. Brit. J. Cancer. 88, 1615–1621. 10.1038/sj.bjc.6600961 12771931 PMC2377124

[B33] Quiles M. T. Arbós M. A. Fraga A. De Torres I. M. Reventós J. Morote J. (2010). Antiproliferative and apoptotic effects of the herbal agent pygeum africanum on cultured prostate stromal cells from patients with benign prostatic hyperplasia (BPH). Prostate. 70, 1044–1053. 10.1002/pros.21138 20503393

[B34] Schwinn D. A. Michelotti G. A. (2000). Alpha1-adrenergic receptors in the lower urinary tract and vascular bed: potential role for the alpha1d subtype in filling symptoms and effects of ageing on vascular expression. BJU Int. 85 (Suppl. 2), 6–11. 10.1046/j.1464-410X.2000.00061.x 10781179

[B35] Shibata K. Katsuma S. Koshimizu T. Shinoura H. Hirasawa A. Tanoue A. (2003). Alpha 1-Adrenergic receptor subtypes differentially control the cell cycle of transfected CHO cells through a cAMP-dependent mechanism involving p27Kip1. J. Biol. Chem. 278, 672–678. 10.1074/jbc.M201375200 12409310

[B36] Shimizu S. Shimizu T. Tsounapi P. Higashi Y. Martin D. T. Nakamura K. (2015). Effect of silodosin, an alpha1A-adrenoceptor antagonist, on ventral prostatic hyperplasia in the spontaneously hypertensive rat. Plos One. 10, e0133798 10.1371/journal.pone.0133798 26308715 PMC4550428

[B37] Strand D. W. Costa D. N. Francis F. Ricke W. A. Roehrborn C. G. (2017). Targeting phenotypic heterogeneity in benign prostatic hyperplasia. Differentiation. 96, 49–61. 10.1016/j.diff.2017.07.005 28800482 PMC5669834

[B38] Suzuki K. Takezawa Y. Suzuki T. Honma S. Yamanaka H. (1994). Synergistic effects of estrogen with androgen on the prostate–effects of estrogen on the prostate of androgen-administered rats and 5-alpha-reductase activity. Prostate. 25, 169–176. 10.1002/pros.2990250402 8084834

[B39] Takei R. Ikegaki I. Shibata K. Tsujimoto G. Asano T. (1999). Naftopidil, a novel alpha1-adrenoceptor antagonist, displays selective inhibition of canine prostatic pressure and high affinity binding to cloned human alpha1-adrenoceptors. Jpn. J. Pharmacol. 79, 447–454. 10.1254/jjp.79.447 10361884

[B40] Unnikrishnan R. Almassi N. Fareed K. (2017). Benign prostatic hyperplasia: evaluation and medical management in primary care. Cleve. Clin. J. Med. 84, 53–64. 10.3949/ccjm.84a.16008 28084985

[B41] Van Asseldonk B. Barkin J. Elterman D. S. (2015). Medical therapy for benign prostatic hyperplasia: a review. Can. J. Urol. 22 (Suppl. 1), 7–17. https://cancerres.aacrjournals.org/content/62/2/597. 26497339

[B42] Xin X. Yang N. Eckhart A. D. Faber J. E. (1997). Alpha1D-adrenergic receptors and mitogen-activated protein kinase mediate increased protein synthesis by arterial smooth muscle. Mol. Pharmacol. 51, 764–775. 10.1124/mol.51.5.764 9145914

[B43] Yang R. Ma Y. X. Chen L. F. Zhou Y. Yang Z. P. Zhu Y. (2010). Antagonism of estrogen-mediated cell proliferation by raloxifene in prevention of ageing-related prostatic hyperplasia. Asian J. Androl. 12, 735–743. 10.1038/aja.2010.24 20473319 PMC3739309

[B44] Zhou Y. Xiao X. Q. Chen L. F. Yang R. Shi J. D. Du X. L. (2009). Proliferation and phenotypic changes of stromal cells in response to varying estrogen/androgen levels in castrated rats. Asian J. Androl. 11, 451–459. 10.1038/aja.2009.28 19483715 PMC3735307

