# Supplementary material for: Piperazine-Derived α1D/1A Antagonist 1- Benzyl-N- (3-(4- (2-Methoxyphenyl) Piperazine-1-yl) Propyl) -1H- Indole-2- Carboxamide Induces Apoptosis in Benign Prostatic Hyperplasia Independently of α1-Adrenoceptor Blocking
Source: Front Pharmacol. 2021 Jan 27;11:594038. doi: 10.3389/fphar.2020.594038 (PMC7873900; doi:10.3389/fphar.2020.594038)
Supplement: Supplementary file 1 [file datasheet1.pdf]

Supplementary Material

Piperazine-derived $\alpha_{1D/1A}$ antagonist 1- benzyl-*N*- (3-(4- (2-methoxyphenyl) piperazine-1-yl) propyl) -1H- indole-2- carboxamide induces apoptosis in benign prostatic hyperplasia independently of α_1-adrenoceptor blocking

Qing Xiao[1†], Qimeng Liu[2†], Ruchao Jiang[1], Kaifeng Chen[1], Xiang Zhu[1], Lei Ma[1], Weixi Li[4*], Fei He[3*], Junjun Huang[1*]

[1] Guangdong Provincial Key Laboratory of Molecular Target & Clinical Pharmacology, School of Pharmaceutical Sciences and the Fifth Affiliated Hospital, Guangzhou Medical University, Guangzhou 511436, PR China

[2] Genetics Laboratory of Obstetrics, The Second Affiliated Hospital of Zhengzhou University，Zhengzhou 450000, PR China

[3] School of Traditional Chinese medicine, Southern Medical University, Guangzhou 510515, PR China

[4] Yunnan University of Chinese Medicine, Kunming 650500, PR China

[†] They contributed equally to this work.

* Corresponding authors. Tel./fax: +86-02037104150. E-mail addresses: huangjunjun1985@gzhmu.edu.cn (JJ. Huang), hefei@smu.edu.cn (F. He), liweixi1001@163.com (WX. Li)

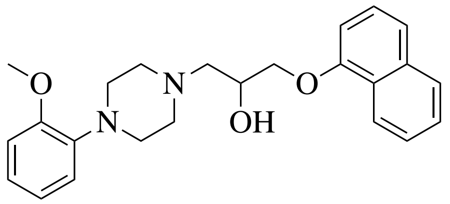

naftopidil (NAF)

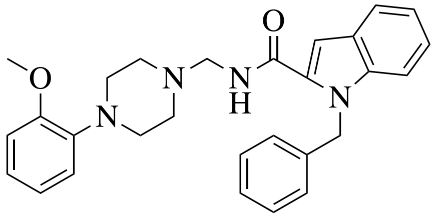

HJZ-12

Supplementary Figure 1 Chemical structures of NAF and HJZ-12

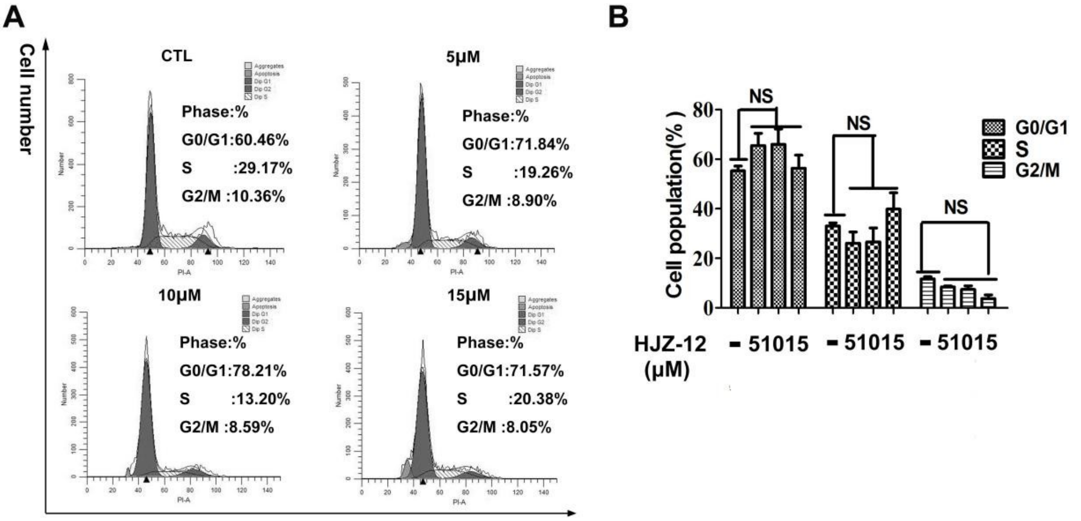

Supplementary Figure 2 Cell cycle analysis of BPH-1 cells treated with HJZ-12 (0, 5, 10 and 15 μM) for 24 h through flow cytometry. The percentages of cells in the G0/G1, S and G2/M phases of the cell cycle were further analyzed using MultiCycle AV software. Significance was determined using one-way ANOVA coupled with Turkey's multiple comparisons test.

Fig. 2B

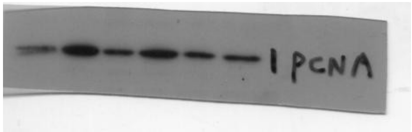

PCNA

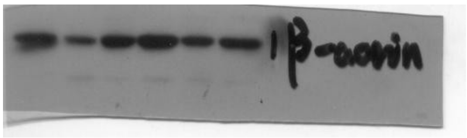

β-actin

Supplementary Figure 3 Original blots shown in the main manuscript. Blots correspond to those shown in Figure 2B within the main manuscript.

Fig. 3A

15 kDa

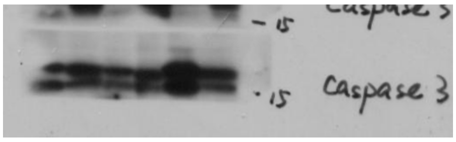

Cleaved-caspase3

55 kDa
40 kDa
35 kDa

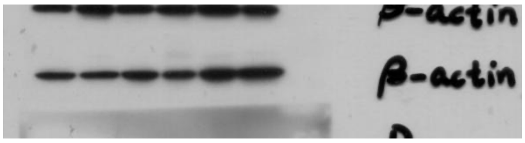

β-actin

Supplementary Figure 4 Original blots shown in the main manuscript. Blots correspond to those shown in Figure 3A within the main manuscript.

Fig. 4B

15 kDa

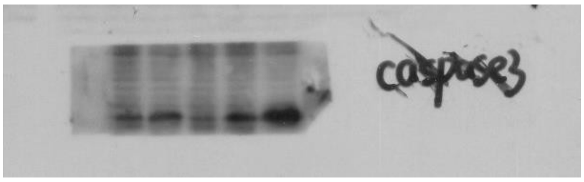

Cleaved-caspase3

55 kDa
40 kDa
35 kDa

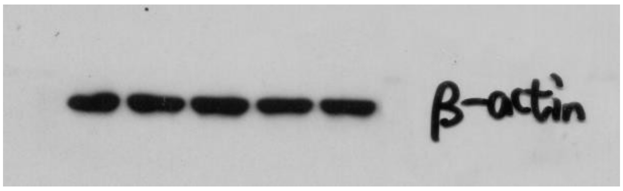

β-actin

Supplementary Figure 5 Original blots shown in the main manuscript. Blots correspond to those shown in Figure 4B within the main manuscript.

Fig. 6C

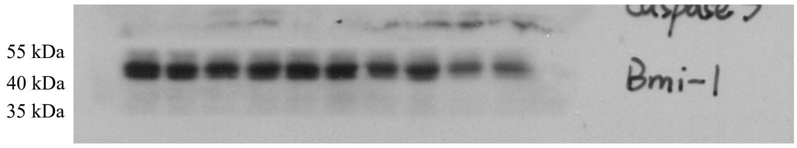

Bmi-1

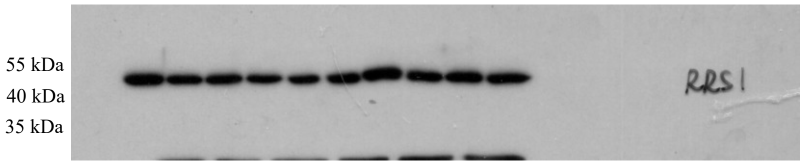

RRS1

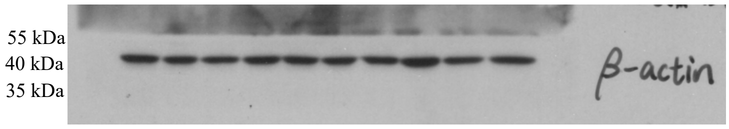

β-actin

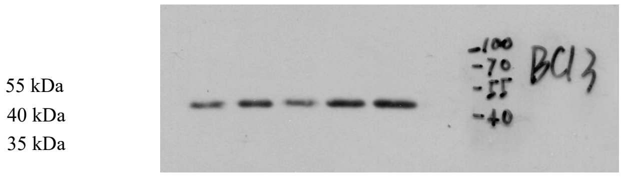

Bcl-3

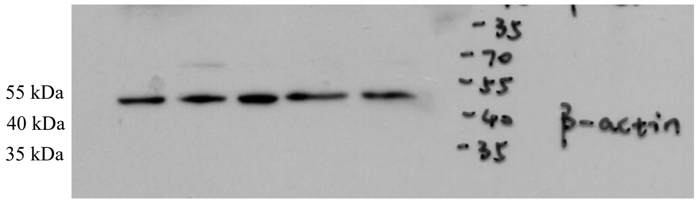

β-actin

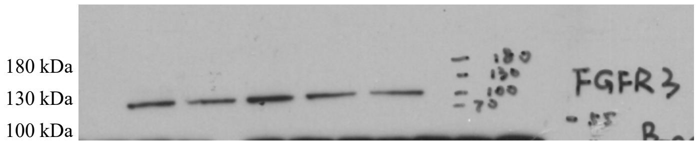

FGFR3

Supplementary Figure 6 Original blots shown in the main manuscript. Blots correspond to those shown in Figure 6C within the main manuscript.

Fig. 7A

55 kDa
40 kDa
35 kDa

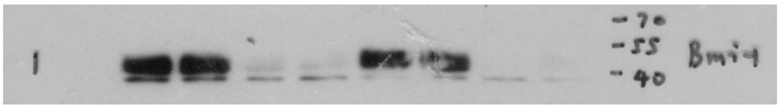

Bmi-1

55 kDa
40 kDa
35 kDa

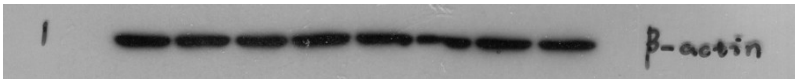

β-actin

Supplementary Figure 7 Original blots shown in the main manuscript. Blots correspond to those shown in Figure 7A within the main manuscript.

Supplementary Table 1 In vitro anti-viability (IC_{50}, μM) of HJZ-12 and NAF in A549 and MCF-7 cell lines

	A549		MCF-7	
	24 h	48 h	24 h	48 h
HJZ-12	5.36	3.35	9.97	8.84
Naftopidil (NAF)	20.14	8.11	46.34	33.85

广州医科大学实验动物伦理委员会审查报告

Committee review of animal experiments in Guangzhou Medical University

项目名称 (Title)	良性前列腺增生动物模型考察候选物药效学实验		
拟申请课题或资金来源 (Project sources)	广州市教育局		
项目申请人 (Applicant)	黄珺珺	受理编号 (Acceptance number)	GY2018-038
审查形式 (Auditing)	函审 Letter correspondence	审查时间 (Processing time)	2018.04.26

审查结果(Results of the review)：

该实验方案符合动物福利伦理要求，通过动物实验伦理审查，准予开展动物实验。

According to the rules of Committee on Animal Research and Ethics, this research project has been reviewed and approved to be appropriate and humane by institutional animal care and use committee.

广州医科大学实验动物伦理委员会

Institutional Animal Care and Use Committee of Guangzhou Medical University

签章(Signature)：[illegible]

时间(time)： 2018.04.26
